# Montmorillonite Exfoliation in LLDPE and Factors Affecting Its Orientation: From Monolayer to Multi-Nano-Layer Polymer Films

**DOI:** 10.3390/polym16020200

**Published:** 2024-01-09

**Authors:** Noémie Rivollier, René Schwiddessen, Geraldine Cabrera, Christelle Combeaud, Susan Schorr, Gilles Dennler

**Affiliations:** 1 Industrial Technical Centre for Plastics and Composites (IPC), 01100 Bellignat, France; 2 Institute of Geological Science, Freie Universität Berlin, Malteserstr. 74-100, 12249 Berlin, Germany; 3 Helmholtz-Zentrum Berlin, Department of Structure and Dynamic of Energy Materials, Hahn-Meitner-Platz 1, 14109 Berlin, Germany; 4 MINES Paris, PSL University, Center for Materials Forming (CEMEF), UMR CNRS 7635, 1 Rue Claude Daunesse, CS 10207, 06904 Sophia Antipolis, France

**Keywords:** montmorillonite, exfoliation, multi-nano-layer co-extrusion, orientation, pole figure

## Abstract

The motivations of the present work are to investigate the exfoliation of montmorillonite within a linear low-density polyethylene matrix and to control its orientation during the cast extrusion process. The first part is focused on the exfoliation of the montmorillonite through the melt extrusion process. The accuracy and relevance of each method used to determine the exfoliation state of montmorillonite have been examined, thanks to X-ray diffraction, transmission electronic microscopy, and rheology. All these methods have presented limitations, but the combination of all leads to a better estimation of the exfoliation state. Finally, the orientation of the montmorillonite is quantified systematically by X-ray texture analysis and correlated with process parameters to discern which one is affecting their orientation. The results have demonstrated an enhancement of the “in-plane” orientation of the montmorillonite with the exfoliation, especially at high concentration and when combined with cast extrusion. Finally, in the multi-nano-layer polymer film configuration, the reduction of the individual layers 29 nm thickness leads to some orientation improvements. However, these improvements are almost at the same level as the concentration effect in a monolayer system. This work gives an overview of all the parameters needed to achieve a significant organo-modified montmorillonite “in-plane” orientation.

## 1. Introduction

Polymer nanocomposites have been the subject of extensive research in the past few decades for a wide range of applications [1,2,3]. Many studies have focused on the addition of 2D nanomaterials into the polymer matrix to make use of their specific shape to enhance the electrical conductivity [4,5], barrier properties toward gas diffusion [5,6,7], or flame retardancy [5,8] of such nanocomposites. One of the most-used 2D materials is montmorillonite (MMT). It is a low-cost, naturally abundant material, which is fairly easy to exfoliate (with an interlayer binding energy of 389.94 kcal.mol^−1^ for the Na^+^ interlayer ion MMT [9]) and to embed in a polymer [10]. Different methods can be employed for these purposes. The most commonly used is in situ exfoliation; the MMT powder is dispersed in a solution containing some monomers [10,11]. The solvent allows the MMT to swell and exfoliate, which releases nanosheets dispersed in the solution. Polymerization is then triggered around the MMT nanosheets. Another route is solution exfoliation [10,12], in which the MMT powder is dispersed in a solvent in which the polymer is soluble. Similar to the previous case, the solvent causes the MMT to swell and exfoliate, and the polymer chains surround the MMT nanosheets. Another exfoliation approach can occur in the solid state through the equal channel angular extrusion (ECAE) technique. Initially, a molten mixture of polymer with montmorillonite is prepared. Once the mixture has solidified, it is introduced into a bent channel with a 90° angle and a cross-sectional area of a few millimeters; then, it can pass through several successive bent channels. This passage through the channels induces significant plastic deformation through shear, thereby promoting the exfoliation of montmorillonite, which subsequently disperses into the polymer matrix [13]. The last commonly used method is melt exfoliation [10,14], during which the MMT is blended with the molten polymer in a mixer or an extruder. The mechanical energy generated by the shear forces overcomes the interactions between the MMT nanosheets. This mechanism can be enhanced by partial intercalation of polymer chains between the individual sheets of the MMT. In some cases, water may be added to assist the exfoliation process [15]. Among all these approaches, melt exfoliation is considered most suitable for large-scale exfoliation. 

As mentioned above, the orientation of the 2D nanomaterials within the polymer matrix is a key parameter for tuning the properties of the polymer nanocomposites [16]. Previous works have shown that cast extrusion is the most effective method of obtaining a mainly parallel orientation of the MMT nanosheets with the sample surface [17]. While the typical thickness of films produced by cast extrusion ranges from a few tens of micrometers to several hundreds of micrometers, multi-nano-layer (MNL) co-extrusion allows one to produce continuous films composed of up to thousands of individual layers for the very same overall film thickness [18]. Thus, each individual layer forming an MNL film can be almost as thin as 2D MMT sheets. This intriguing observation suggests that producing an MNL film from an MMT nanocomposite may significantly enhance the orientation of 2D MMT sheets compared to what is achievable by simple cast extrusion. 

In fact, a few studies have combined MMT-based nanocomposites with the multi-nano-layer polymer co-extrusion approach in order to improve the gas barrier properties of polymer films [19,20,21]. Unfortunately, only very small effects could be observed, and the orientation of the MMT was neither quantified nor correlated with process conditions.

Thus, the work presented here aims to understand the phenomena affecting the orientation of exfoliated MMT nanosheets embedded in a polymer matrix processed via MNL co-extrusion. We chose to work with a linear low-density polyethylene in which the MMT was exfoliated by the melt extrusion process. A detailed description of the methods used to characterize the exfoliation of MMT is provided, as few articles focus on the combination of all the techniques we have employed, and each of the methods has limitations that may influence the characterization of exfoliation levels, especially in a non-polar polymer matrix. Finally, we have thoroughly studied and quantified the orientation of the MMT nanosheets by X-ray texture analysis, and correlated these results with different process parameters and film architectures. 

## 2. Materials and Methods

Linear low-density polyethylene (LLDPE) was supplied from Dow Chemical, Midland, MI, USA (DOWLEX 2645™). It has a melt flow index (MFI) of 0.85 g/min under 2.16 kg at 190 °C, a density of 918 kg/m^3^ at 27 °C, and a molecular weight of 108,780 g/mol [22]. Anhydride maleic grafted linear low-density polyethylene (LLDPE-g-MA) was purchased from SK Functional Polymers, Courbevoie, France (OREVAC^®^ 18341) as a compatible material for LLDPE and montmorillonite. It has an MFI of 1.50 g/min under 2.16 kg at 190 °C, and a density of 920 kg/m^3^ at 27 °C. Two different montmorillonites (MMT) were purchased from BYK, namely Cloisite Na^+^ (CNa^+^) and Cloisite 20A (C20A). The former one has Na^+^ cations only as intercalating agents between two MMT monolayers, while the latter one is intercalated with ammonium organic modifier cations composed of two long alkyl chains of 16 carbons and two methyl groups as intercalating agents [23]. In addition to increasing the interlayer space and reducing interactions between the MMT monolayers, functionalization promotes excellent compatibility with a polyolefin matrix. Given that the MMT is polar, the apolar properties of the functionalized ion encourage the intercalation of non-polar chains between the MMT nanosheets, thereby facilitating the subsequent exfoliation process. MMT monolayers are composed of a tetrahedral–octahedral–tetrahedral (TOT) layer, resulting in a monoclinic structure with the space group C2/m, with C for the base face-centered lattice, and 2/m for the mirror plane symmetry. The unit cell has the following dimensions: α = γ = 90°, β = 94.48° a = 5.163 Å and b = 8.986 Å. The lattice parameter and c are variable depending on the distance between the TOT layers and therefore the intercalated ion.

Before compounding the various batches, the two montmorillonites and the LLDPE-g-MA were dried under vacuum at 80 °C for 24 h. All compounds were made with a 2:1 ratio of LLDPE-g-MA: MMT and melt-blended with a co-rotating twin-screw extruder of 18 mm diameter (ZSK 18 MEGAlab from Coperion, Stuttgart, Germany) with an L/D ratio of 40 and a high shear extrusion profile with two zones containing kneading elements, and the rest is convoying elements. The temperature of extrusion was set at 190 °C, while the screw speed was set at 500 rpm. For both montmorillonites, the master batches with 20 wt% of montmorillonite in LLDPE were first compounded with a feed rate of 5 kg.h^−1^. Batches with 5 wt% MMT were produced by further diluting the master batches with LLDPE at various feed rates ranging from 0.5 to 10 kg.h^−1^. Two additional dilutions were compounded from C20A master batch to obtain 10 wt% and 15 wt% dilutions, with a feed rate set at 5 kg.h^−1^. A list of the compounds and theirs name is presented in Table 1 below. 

To determine the factors affecting the orientation of the montmorillonite, we used different three processes detailed below to process the previous compounds in the melting state. 

Thermo-compressed films of about 100–150 µm thickness were produced with a Hydraulic Carver Press under 6000 Lb at 190 °C. A list of the films is presented in the Table 2 below.

Monolayer films of 100 µm thickness were made by cast extrusion at 200 °C with a mono-screw extruder (E45 from Collin (Maitenbeth, Germany), 25 L/D) at a feed rate of 5 kg/h. A list of the films is presented in the Table 3 below.

**Figure 1:**
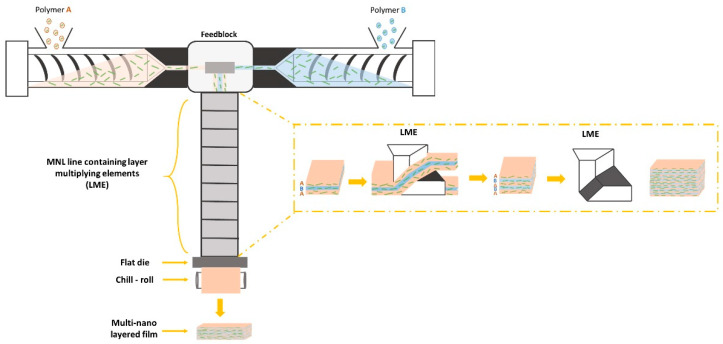
Scheme of the multi-nano-layer co-extrusion process; the extruder has a 25 L/D ratio.

The multi-nano-layer co-extrusion process allows us to produce films composed of up to a few thousand individual layers within a single step process (Figure 1). These layers can be as thin as a few tens of nanometers for an overall film thickness of several tens of micrometers [18,24]. This technique relies on the use of multiplying elements that induce an alternation of two different polymer phases, *A* and *B*, chosen to be of the same nature or not. The thickness of the individual layers is calculated using the following equation:

(1) $$h_{individual\;layer\;A\;or\;B}=\varphi_{A\;or\;B}\times \frac{h_{film}}{2^{n+1}+1}$$

where *h* is the thickness of the overall film, *φ* is the proportion of phase A or B, (2^*n*+1^ + 1) is the number of layers in the film, and *n* is the number of layers multiplying elements. 

**Figure 2:**
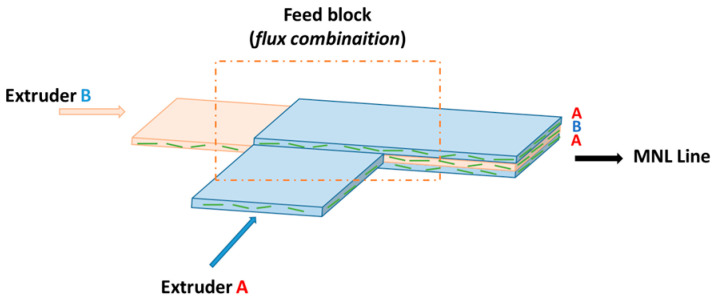
Scheme of the combination of polymer A and B flux in the feed block.

To manufacture MNL films, two single-screw extruders (E30E and E45E from Collin, featuring a 30 mm and 45 mm diameter, respectively, and a 25 L/D ratio for both) were connected by a co-extrusion feed block (Nordson), which combines polymer melting flux together within an A/B/A configuration. Indeed, the feed block consists of several channels. There is a central channel wherein extruder B is connected, and a lateral channel wherein extruder A is connected. The flow from extruder A is then divided into two to go on either side of the central flow, which is composed of polymer phase B. Thus, in this configuration, there is a three-layer flow (A-B-A) at the output of the feed block, which will then go through the MNL line (Figure 2). Two film architectures were produced: one with phase A using only LLDPE, and phase B with 5 wt% of MMT (C20A_5_5 blend) to verify the layer architecture; the other with both phase A and B equivalent, containing 5 wt% of MMT. The flow ratio of A/B phases was set at 50/50. 

**Figure 3:**
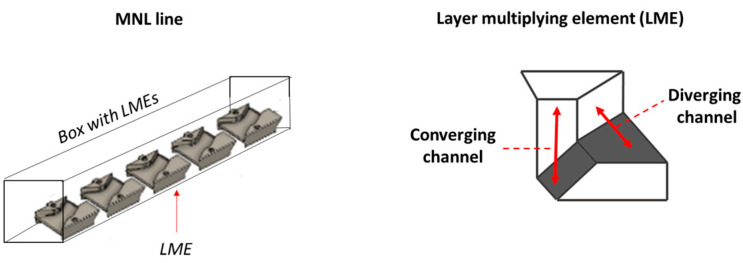
Scheme of the LME disposition in the MNL extrusion line. Scheme of the layer-multiplying element with the identification of converging and diverging channels.

Different numbers of layer-multiplying elements (LME) were added in the MNL extrusion line (Nordson), namely 0, 3, 6, 8, and 9. The role of an LME is to vertically divide the polymer flow, allowing each stream to pass on either side of the multiplier element. Within the element, the flow is initially horizontally compressed in a converging channel and then horizontally stretched in a diverging channel (Figure 3). Finally, the two streams are horizontally recombined at the output of the multiplier element. As a result, the number of layers has been multiplied (Figure 1) [25]. In the MNL line, they are aligned one after the other (Figure 3). The length of the MNL line is always the same, regardless of the number of multiplier elements. Therefore, when the number of multiplier elements decreased, we chose to space them evenly within the MNL line. 

These films were all extruded at 200 °C and at 5 kg/h in the MNL line down to the 400 µm slot die, after which they were cooled down to 90 °C on a chill roll. The speed of the chill roll was varied from 3 to 10 m.min^−1^, which allowed us to more or less stretch the films and reduce their overall thickness from 800 µm (due to the thermal expansion of the polymer at the exit of the open die at 400 µm) to 30 µm. The stretching speeds correspond to a draw ratio (Dr) ranging from 6 to 23. A list of the manufactured films is presented in the Table 4 below.

Bi-axial stretching was carried out using ETIFI equipment designed by the CEMEF. A 24 × 24 mm sample was heated to 90 °C, which is below the LLDPE melting temperature, and concurrently stretched in two directions at a rate of 1 mm/s until achieving a deformation of 7 × 7. The sample was subsequently cooled with air to solidify the microstructures formed during the bi-axial stretching process. 

The X-ray diffraction (XRD) and texture analysis process was carried out as follows.

XRD measurements were carried out at a Bruker D8 diffractometer in reflexion mode using Bragg Brentano configuration at room temperature. Patterns were recorded with a 0.0158° step size and along a 2θ angle range from 0 to 65° with a 40 kV, 40 mA. A CuK_α_ radiation source was used, with a wavelength λ = 1.5406 Å (CuK_α1_). A beam knife was used to cut off one part of the incident X-Ray beam to prevent the primary beam from hitting the detector at angles below 5°.

Texture analysis was performed using a PANalytical MRD X-ray diffractometer equipped with an Eulerian cradle employing two-axis scans along φ and χ. The intensity distribution for a specific 2Ɵ angle corresponding to the studied lattice plane was recorded along φ from 0° to 360° and at sample tilts χ from 0° to 85°, with an increment of 5° each. All values of φ and χ were plotted on a pole figure graph, where all intensity values were normalized by dividing them by the mean of the entire set of intensity values. This allowed us to obtain a multiple over random distribution (MORD) value, facilitating a reliable comparison regardless of the concentration of MMT.

A 40 kV, 40 mA CuK_α_ radiation source was used, with a wavelength λ = 1.5406 Å (CuK_α1_). The sample size was a square of 5 × 5 cm. The films were adhered to a glass substrate to ensure the flattest possible surface and to avoid any impact on orientation measurements.

Rheology:

Small amplitude oscillatory shear measurements (SAOS) were carried out with a stress-controlled rotational TA instrument, New Castel, DE, USA AR2000ex rheometer with a 25 mm parallel plate geometry. All measurements were performed within the linear viscoelastic domain at 0.08% of deformation. Dynamic frequency sweep tests were measured over an angular frequency range from 0.08 to 628 rad.s^−1^ at 170 °C.

Optical microscope (OM):

Optical microscope observations were performed with a Keyence, Osaka, Japan VHX 7000, in transmission mode and with a polarized light for the MNL samples. To assess the exfoliation of MMT, cross-sections of 10 µm were cut from extrudate samples using a microtome. For characterizing the layered morphology of MNL films, 10 µm thick cross-sections were obtained from unstretched MNL films with an initial thickness of 800 µm, using a microtome. This film thickness facilitates the observation of layers, but also aids in the preparation of the samples.

Transmission electronic microscope (TEM):

TEM observations were carried out on a 30 nm slice from extrudates, cut with a cryo-microtome. A JEOL, Tokyo, Japan 1400 Flash transmission electronic microscope was used at 120 kV and ×8000 magnification.

## 3. Results and Discussion

In the first part, we focused on the process conditions necessary for the exfoliation of montmorillonite in our system to achieve the best possible exfoliation. Once exfoliation was achieved, we aimed to study the parameters influencing the orientation of montmorillonite nanofillers in systems with a simple monolayer-type architecture. Subsequently, we sought to investigate the impact of a more complex film architecture on the orientation of these fillers using the multi-nano-layer co-extrusion technique.

### 3.1. Characterization of the In-Situ Exfoliation of MMT Nanosheets

The mechanical exfoliation of MMT uses the shear forces generated by the twin-screw extruder to overcome the Van der Waals interactions between the MMT nanosheets [14]. This allows us to obtain an exfoliated morphology directly in the polymer melt. The exfoliation is affected by several process parameters that contribute to the specific mechanical energy brought by the latter [26,27]. Vergnes et al. [28] have studied the impact of twin-screw speed, feed rate, and screw profile on the exfoliation of MMT in a polyolefin matrix. They showed that a high shearing screw profile is necessary to achieve effective exfoliation, and that from a certain screw speed on, exfoliation is no longer improved with increasing screw speed. Moreover, they evidenced that the residence time of the polymer melt along screws is the most important parameter to control the exfoliation effectiveness [29]. Thus, they concluded that for a constant screw speed, the slower the feed rate, the more complete the exfoliation.

In order to optimize the exfoliation of the two MMTs and compare their respective capacities for exfoliation, the influence of the feed rate upon the level of exfoliation of the MMT dispersed in our system has been investigated. For that purpose, we implemented several characterization techniques, namely XRD, rheology, and microscopy. The accuracy and relevance of each of these methods were discussed. Finally, we also investigated the influence of the exfoliation state on the “in-plane” orientation of the MMT.

#### 3.1.1. Determination of the 00*l* Bragg Peak Positions via XRD

Most of the studies that have investigated MMT–polymer nanocomposites have used XRD to tentatively characterize the exfoliation status of the MMT [5,30]. Usually, the position of the 001 or 002 Bragg peak of the MMT is followed, as it corresponds to two MMT nanosheets lying parallel to each other. The position of these peaks depends on the interlayer distance between lattice planes. In the case of an intercalation of polymer chains between the MMT nanosheets, the distance increases, and the Bragg peaks shift to smaller 2θ angles. For complete exfoliation, the absence of a diffracting plane results in the disappearance of the MMT 00*l* Bragg peak [31].

**Figure 4:**
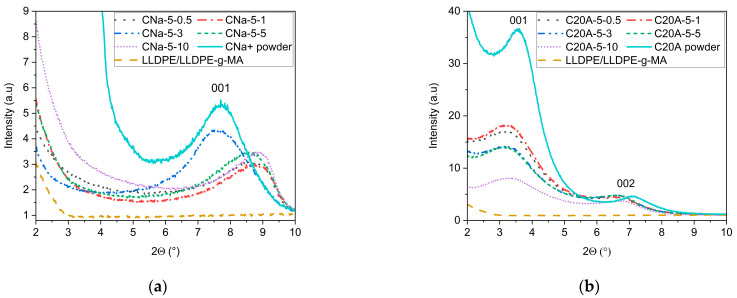
BBXRD diffractograms showing the impact of the feed rate on the positions and intensities of MMT 001 and 002 Bragg peaks. (**a**) Na^+^ montmorillonite-based compounds. (**b**) C20A montmorillonite-based compounds.

Figure 4a,b show the XRD patterns of all the compounds listed in Table 1 made with 5 wt% of CNa^+^ and C20A, respectively and the LLDPE/LLDPE-g-MA matrix alone. The matrix alone does not exhibit diffraction peaks below 10°, ensuring that the Bragg peaks observed in samples with montmorillonite belong to it. In both cases, the variations in the extrusion feed rate do not result in the disappearance of the MMT 001 or 002 Bragg peaks. In the case of the CNa^+^ MMT, the pristine powder shows a 001 Bragg peak at 2θ = 7.7° while most of the compounds exhibit a 001 Bragg peak at almost 9°. This shift to a larger angle suggests a reduction in the distance between the basal planes of 170 pm. We believe that this may be due to the removal of Na^+^ ion during the process, as this ion has a diameter of about 200 pm [32]. 

Looking at the diffraction patterns of the C20A compounds, one can note a slight increase in the distance between basal planes compared to the pristine powder (i.e., a slight shift of the 001 Bragg peak towards smaller 2θ angles). This may stem from the intercalation of LLDPE chains between the MMT nanosheets instead of, or in addition to, the initial organo-modified interlayer ion [23,33]. The position of the 001 Bragg peaks is quite similar for all feed rates, but their intensities do vary. These variations might be related to a decrease in the quantity of 00*l* diffraction planes in diffraction conditions induced by the exfoliation [5,33]. But this interpretation has to be taken with great caution; indeed, at 2θ angles below 5°, the contribution of the direct beam can be sizable and may interfere with the actual contribution of the diffraction planes. Thus, we conclude that it is not possible to evaluate the state of exfoliation of the compounds using conventional XRD.

#### 3.1.2. Quantification of Melt Yield Stress (σ_0_) by Rheology

Rheology is not a direct and systematic method of characterizing the state of exfoliation of montmorillonite, yet it can provide a pertinent overview of the state of exfoliation of MMT within polymer-based compounds [30]. Indeed, the exfoliation of the 2D material can lead to the creation of a network of MMT nanosheets. These objects may interact mechanically and thereby influence the complex viscosity measured at low frequencies [34]. This specific behavior is described by the Carreau–Yasuda model [35] expressed in Equation (2), where the yield stress *σ_0_* is correlated with the exfoliation level of MMT nanosheets [36]:

(2) $${ƞ}^{*}\left(\omega \right)=\frac{\sigma_{0}}{\omega }+{ƞ}_{0}{\left[1+{\left(\lambda \omega \right)}^{a}\right]}^{\frac{n-1}{a}}$$

where ƞ* is the complex viscosity, *σ_0_* is the melt yield stress, *ω* is the angular frequency, *ƞ*_0 is_ the Newtonian viscosity, *λ* is the characteristic relaxation time, *a* is the Yasuda parameter, and *n* is the shear-thinning index.

**Figure 5:**
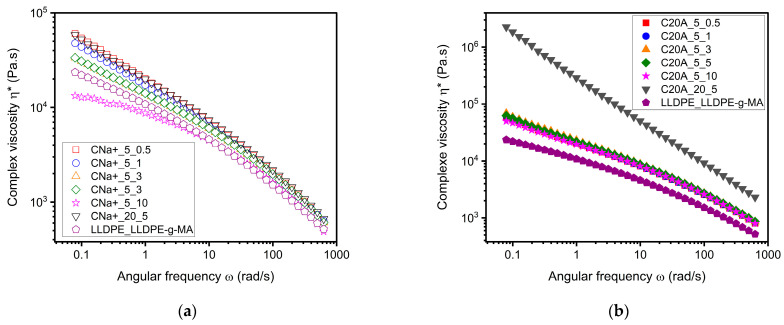
Dynamic frequency sweep measurements at 190 °C showing the impact of the feed rate on the complex viscosity |ƞ*|. (**a**) Compounds with Na^+^ montmorillonite. (**b**) Compounds with C20A montmorillonite.

Figure 5a,b display the complex viscosity of compounds with both MMTs, as well as that of the pristine polymer. They reveal several noticeable features. First, one can note that the addition of fillers increases the complex viscosity of the compounds compared to that of the polymer matrix alone over the entire frequency range. There is also an increase in the complex viscosity when the filler concentration is increased to 20 wt%, especially for the C20A MMT. This phenomenon is quite intuitive, as the MMT is an inorganic material with much higher mechanical properties than those of LLDPE. More interestingly, Figure 5a shows some differences in viscosity at low frequencies for the different CNa^+^ compounds. The related melt yield stresses, σ_0_, calculated from Equation (2), are indicated in Table 5 and Table 6. Their values reduce significantly with increasing feed rates. According to Vergnes et al. [23], this suggests that the state of exfoliation of CNa+ decreases with increasing feed rate. This is because with a high feed rate, the residence time is reduced, giving the MMT less time to be exfoliated along the screws.

In the case of the C20A compounds (Figure 5b), no variations in viscosity can be observed for mixtures with 5% MMT prepared at various feed rates. The calculated melt yield stresses listed in Table 6 show values that are quite close to each other. This suggests that these compounds reached their maximum state of exfoliation independently of the feed rate. Indeed, thanks to the intercalation of chains between the nano-layers, interactions are reduced, and less energy is required from the process to achieve a good level of exfoliation. Therefore, it is assumed in this case that the minimum energy supplied by the highest flow rate is already sufficient to exfoliate the montmorillonite. Additionally, the melt yield stresses of the 5 wt% C20A compounds are at least two times larger than the compounds with CNa^+^ prepared under the very same conditions. We believe that this stems from the fact that higher exfoliation states yield larger mechanical interactions between the 2D nano-sheets. This is further confirmed by the value of σ_0_ obtained for the compound composed of 20 wt% of C20A MMT, which is about 67 times larger than the one calculated for the compound composed of 20 wt% of CNa^+^ MMT. Hence, we can conclude that the compounds with CNa^+^ MMT were probably not completely exfoliated, while the ones with C20A MMT were likely to be almost entirely exfoliated.

Thus, complex viscosity measurements combined with the Carreau–Yasuda model allow us to gain some insight into the level of exfoliation of the MMT. This method provides significantly more information compared to the previously discussed XRD patterns, which do not offer detailed insights into the state of the 2D material embedded within the polymer matrix.

#### 3.1.3. Morphology Characterization by Microscopy

Microscopy is another technique widely used to characterize the state of exfoliation of MMT in polymer nanocomposites [35]. It offers a direct observation of the morphology of the compounds, but has the drawback of focusing only on a very small part of the samples.

**Figure 6:**
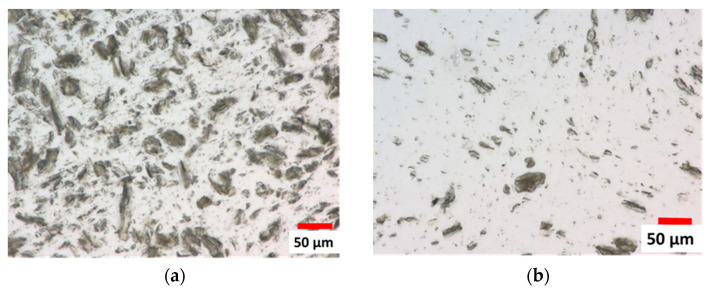

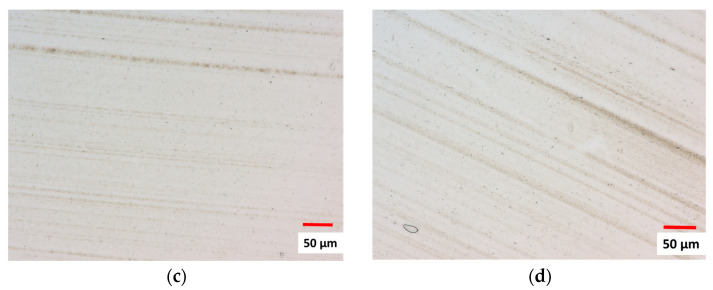
Optical microscope images of strand cross-sections in transmission mode. (**a**) CNa-20-compound; (**b**) CNa-5-5 compound; (**c**) C20A-20-5 compound; (**d**) C20A-5-5 compound.

**Figure 7:**
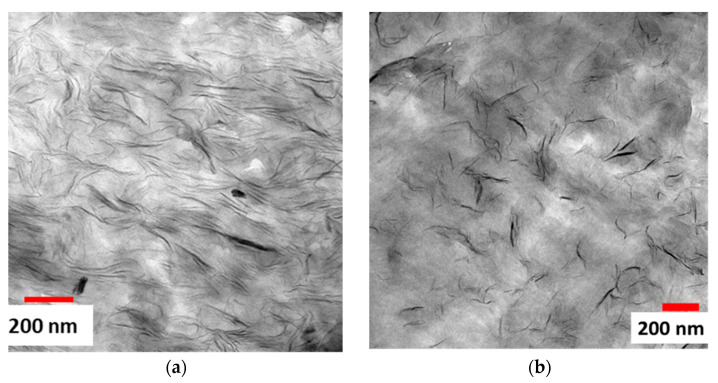
TEM observations of strand cross-sections: (**a**) C20A-20-5 compound; (**b**) C20A-5-5 compound.

Figure 6a,b show optical microscope images of compounds’ strand cross-sections with 5 and 20 wt% of CNa^+^ MMT at 5 kg.h^−1^, respectively. One can see that the MMT particles do not seem exfoliated, and appear as tactoïd agglomerates. In contrast, Figure 6c,d show a thinner dispersion in the compounds containing 5 and 20% C20A MMT, respectively. Therefore, to be able to identify any exfoliation of the C20A MMT, more powerful microscopic analyses are needed. Indeed, the TEM images in Figure 7a,b, reveal that the major parts of montmorillonites are exfoliated in compounds containing 5 and 20% C20A MMT, respectively. Stacks of a few nanosheets are still visible, but with an overall thickness of a few tens of nanometers, which is drastically different from the situation shown in Figure 6a,b. Thus, these observations do confirm the conclusions drawn from the above complex viscosity measurements: compounds with the CNa^+^ MMT are not exfoliated, while those with the C20A MMT are mainly exfoliated with nano-sheets 200 nm in length and 1 to 10 nm in thickness.

#### 3.1.4. The Impact of the Exfoliation State on the Orientation of the MMT

We also investigated the impact of the exfoliation state upon the orientation of the MMT nanosheets. As demonstrated previously, compounds with the CNa+ MMT do not show a large level of exfoliation, while C20A-based compounds do. Therefore, we compared the crystalline orientation of the MMT for the C20A and CNa^+^ mixtures prepared with the same concentration of MMT, namely 5 wt%. The samples are monolayer films made by thermo-compression. 

**Figure 8:**
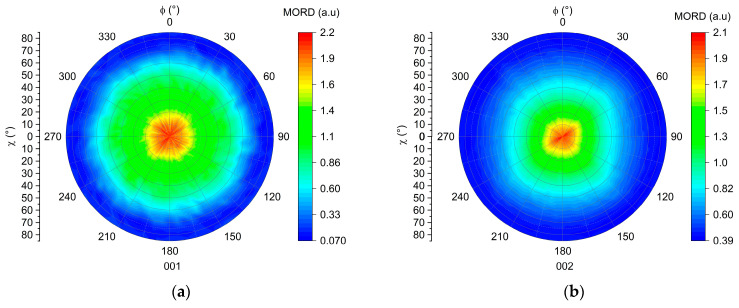
(**a**) Pole figure of the (001) lattice plane of the MMT in the CNa-5-5 compound. (**b**) Pole figure of the (002) lattice plane of the MMT in the C20A-5-5 compound.

XRD texture analysis were carried out to plot the pole figures of the (001) for the CNa^+^ MMT and (002) for the C20A MMT lattice planes [37,38]. The results, shown in Figure 8, gather the MORD intensities of the 001 Bragg peak investigated as a function of the χ and Φ positions. The χ angle corresponds to the inclination angle of the sample during the measurement, and Φ encompasses all directions around the film. Thus, we are able to measure the intensity diffracted from the lattice plane, under investigation for each χ and Φ position [39].

Figure 8a,b exhibit both a diffraction maximum at the center of the pole figure. This indicates that most of the MMTs have the normal to their (001) or (002) lattice planes oriented with an χ angle between 0 and 10° compared to the normal of the film. Thus, the majority of the montmorillonite nano-sheets are oriented “in-plane” in both films. However, in Figure 8a, the diffraction intensity distribution is broader than in Figure 8b. This indicates that the (001) lattice planes of the CNa^+^ MMT nano-sheets are oriented with χ angles up to 60° from the film normal, while χ does not reach values higher than 40° in the case of C20A MMT. We can thereby conclude that the CNa^+^ MMT nano-sheets are less oriented “in-plane” than the C20A MMT. Thus, these results evidence a significant impact of the exfoliation state upon the overall orientation of MMT within the compounds. Due to their 2D shape, the exfoliated MMT nano-sheets tend to orient more in the direction of the flow of the melted polymer [17]. On the contrary, when the MMTs are in the tactoïd state, they have a lower aspect ratio and are less influenced by flow of the melted polymer. 

In this part, results have demonstrated that the combination of all techniques is necessary to draw accurate conclusions about the exfoliation state. In this case, the melt yield stress serves as the best indicator of the level of exfoliation, while XRD is essential for assessing complete exfoliation; microscopy is used to confirm all the hypotheses. The comparison of the two types of montmorillonite reveals that C20A MMT is the most suitable for achieving an exfoliated morphology in a LLDPE/LLDPE-g-MA blend.

So, for the rest of the study, the C20A MMT compound manufactured with a feed rate of 5 kg.h^−1^ was chosen. It shows a good exfoliation state and a good ability to be oriented “in-plane”, and the feed rate is reasonable for medium-scale production. 

### 3.2. Factors Influencing the Orientation of the MMT in a Monolayer Film

After exfoliating the MMT, our goal is to orient the 2D nano-sheets parallel to the film surface. Due to their 2D plate shape, the MMT nano-sheets tend to orient themselves in the flow direction during the manufacturing process [17]. Furthermore, we have previously demonstrated that they tend to exhibit a more “in-plane” orientation when MMT is exfoliated. However, this orientation might be enhanced by other parameters. Therefore, we explored several factors that might affect the orientation of the MMT nano-sheets in monolayer films, such as the concentration of MMT and the manufacturing process of the polymer film.

#### 3.2.1. The Concentration of Montmorillonite

**Figure 9:**
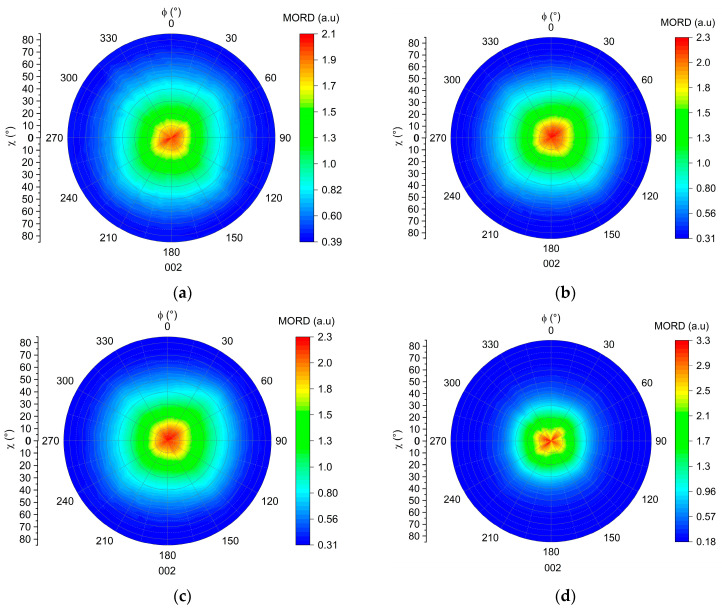
Pole figures of the (002) lattice plane of the MMT nano-sheets embedded in films prepared by the thermo-compression process: (**a**) C20A-5-5 compound; (**b**) C20A-10-5 compound; (**c**) C20A-15-5 compound; (**d**) C20A-20-5 compound.

We studied first the influence of the MMT concentration in the mixtures. For this purpose, films containing 5, 10, 15 and 20 wt% of C20A MMT were prepared by thermo-compression. The orientation of the MMT in these films was measured as previously explained, and is reported in the pole figures shown in Figure 9. 

**Figure 10:**
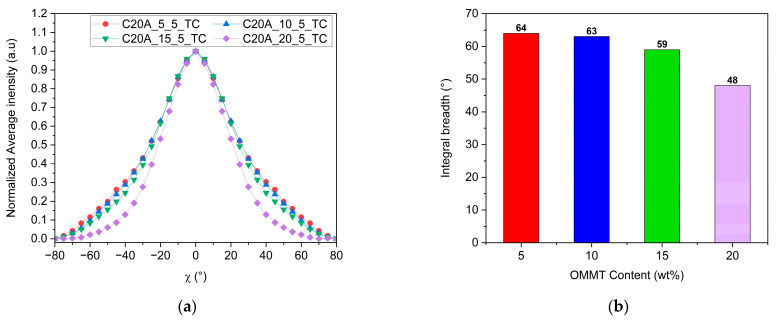
(**a**) Average intensity over Φ versus χ from 002 MMT pole figures in Figure 9. (**b**) Calculated integral breadth of peaks in (**a**).

One way to quantify the orientation of the MMT is to use the Herman’s parameter extracted from the pole figures [40,41]. However, while this parameter seems suitable for determining perfect “in-plane” or “on-edge” orientations, it appears inappropriate in our case, as we are attempting to find evaluable differences between samples in which the crystalline orientation is more random. Therefore, we chose to call upon another method to quantify the “in-plane” orientation of the MMT nano-sheets in our samples. This is based on the characterization of the width of the signal, achieved by plotting the normalized average intensity of the XRD signal measured at a given Φ angle and over χ (Figure 10a), and by calculating the so-called integral breadth according to the following equation (Figure 10b):

(3) $$Integral\;breadth\;\left({}^{\circ}\right)=\frac{Peak\;area}{Peak\;height}$$

According to Equation (3), the sharper the peak, the smaller the dispersion around 0° along χ, and the smaller the integral breadth. At a concentration of 5 and 10 wt% of MMT, the orientation is similar, with integral breadth values of 64 and 63°, respectively. With the increase in the concentration up to 15 and 20 wt%, the integral breadth decreases to 59° and 48°, respectively.

We can thereby conclude that after reaching a certain MMT concentration threshold (between 15 and 20 wt%), the “in-plane” orientation of the MMT nanosheets is significantly increased. This is consistent with the observations made in Figure 5b. Rheological measurements indicated more interactions at 20 wt% of MMT, which might also involve particle–particle interactions in addition to polymer–particle interactions. As is also shown in Figure 7a, the extrudate cross-section observation shows that MMT nanosheets are already closer to each other compared to the extrudate with only 5 wt% of MMT. Therefore, in a monolayer film, the thickness of the samples is reduced to 150 µm, and we can assume that the MMT nanoplatelets are closer to each other. Their close packing and interactions may favor ‘in-plane’ orientation via self-assembly mechanisms when the MMT concentration increases [42].

#### 3.2.2. The Manufacturing Process

**Figure 11:**
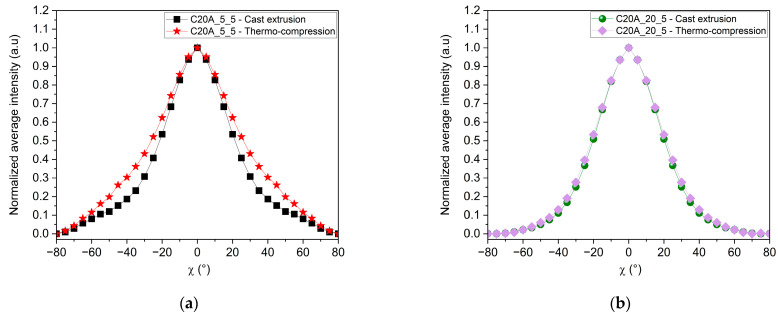
Average intensity over Φ versus χ from 002 MMT pole figures of each sample; comparison between monolayer films made by thermo-compression and cast extrusion: (**a**) 5wt% of MMT and (**b**) 20wt% of C20A MMT.

A third parameter likely to affect the MMT orientation within the polymer matrix is the manufacturing process employed to produce films. Some studies have previously shown that in the case of MMT [40] and 2D materials in general [43], the manufacturing process has an impact on the improvement of the “in-plane” orientation due to the forces generated by the process [44]. Indeed, blow extrusion leads to a better “in-plane” orientation than thermo-compression [23]. In the case of coating deposition, both slot die and spin-coating result in a better “in-plane” orientation compared to drop-casting or processes that do not involve shear forces [43,44]. Thus, we compared the thermo-compression process with monolayer cast extrusion. In the first case, a constant pressure is applied to the molten compound for a few minutes. In the case of cast extrusion, forces are applied for few seconds only as the melt passes through the flat die, but the melt is also subject to shear forces [45,46]. Figure 11 shows the normalized intensity of the XRD signals measured for the 002 Bragg peak of the MMT in the case of films made by the two processes and at two different concentrations, 5 and 20 wt%. Table 7 reports the integral breath extracted in each of these cases.

One can note from Figure 11 that “in-plane” orientation is stronger in 5 wt% MMT films prepared by cast extrusion than in those produced by thermo-compression. This observation is consistent with previous studies that have shown that during the extrusion process, MMT nanosheets tend to orient themselves in the direction of the flow of the melted polymer [17,40] that is parallel to the surface of the film. This phenomenon is mostly due to shear forces present in the cast extrusion die [47]. Interestingly, the influence of shear flow on the “in-plane” orientation appears to be reduced at higher MMT concentrations, as the orientation of the MMT seems similar in both processes at 20 wt%. 

This is consistent with the rheological results obtained in Figure 5b. Indeed, the viscosity is significantly increased, leading to a loss of chain mobility due to the numerous interactions between the fillers and the LLDPE. Consequently, the addition of more MMT nanoplatelets encourages more particle interactions. Rheological investigations into the behavior of nanocomposites with nanoclay reveal that with increasing concentration, they display a Newtonian behavior at low shear rates, transitioning to a shear-thinning behavior at higher shear rates due to the orientation of nanoplatelets in the flow direction [48,49]. In our specific scenario, the shear rate may be insufficient to improve the orientation of MMT in the cast extrusion process.

Thus, the montmorillonite concentration within the polymer matrix and the fabrication process of the films all demonstrated a noticeable impact on the “in-plane” orientation of the MMT in monolayer film configurations. To further improve the orientation, we have investigated the impact of employing a more complex film architecture such as multi-nano-layer co-extrusion. Based on our previous observations, we decided to work with a compound made of 5 wt% of the C20A MMT because of its exfoliated structure and ability to be aligned by shear flow.

### 3.3. Factors Influencing the Orientation of the MMT in Multi-Nano-Layered Films

MNL co-extrusion has been employed in several studies that aimed to tune the final properties of films [21,50] by controlling the local crystallization of polymers [50]. This is possible when the individual layers reach thicknesses as small as a few nanometers; the polymer chains are then locally confined, leading to controlled crystallization. This phenomenon is enhanced when an amorphous polymer A is combined with a semi-crystalline polymer B in an A-B-A configuration. Reducing the layer thickness of the semi-crystalline polymer B to a few nanometers (constrained by the amorphous phase A on both sides) forces its crystals to orient within the layer, triggering a transition from 3D to 2D crystals [51]. 

Based on this principle, we anticipated that embedding 2D nano-objects like MMT nanosheets in a polymer matrix, to be processed by MNL coextrusion, would allow us to force their orientation parallel to the surface of the film. We expected this effect to be especially strong when the thickness of individual layers approached the average lateral size of the exfoliated MMT sheets (≈200 nm, see Figure 7b). In order to verify our hypothesis, we prepared a series of multi-nano-layer films with different number of layers, keeping an overall film thickness of 100 µm. Each layer was made out of a 5 wt% C20A MMT compound (alike above), and were intended to have the same thickness (s ratio of 50/50 between phases A and B, both being equal).

#### 3.3.1. Influence of the Number of Layers

**Figure 12:**
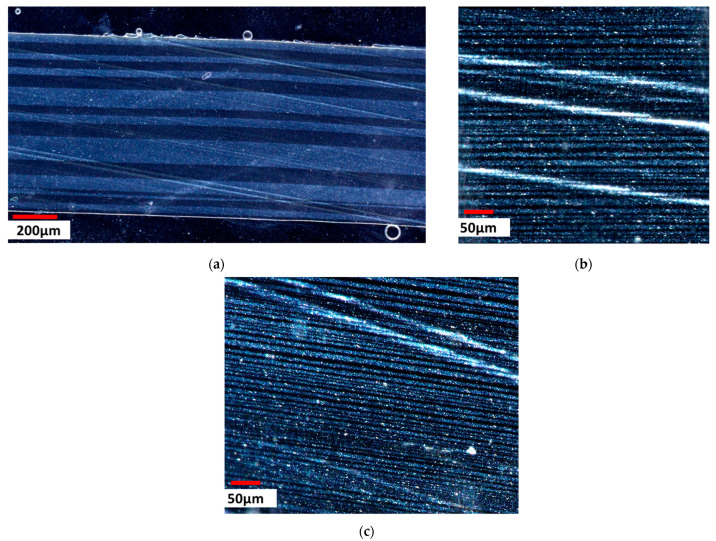
Optical microscope images of MNL film cross-sections. Alternation of a rows of LLDPE and C20A-5-5 compounds: (**a**) 17 layers; (**b**) 512 layers; (**c**) 1025 layers.

Increasing the number of layers in multi-nano-layer films of an identical overall thickness should lead to a reduction in the thickness of the individual layers [52]. In order to confirm that our MNL co-extrusion process produced genuine MNL films, we prepared thick film to be observed with an optical microscope. In order to guarantee a sufficient contrast between adjacent layers, we used a phase A, consisting of MMT nanosheets embedded in an LLDPE matrix, and a phase B, made of virgin LLDPE. Figure 12a–c display cross-sections of films comprising 17, 512 and 1025 layers, respectively. 

As shown in Figure 12, all the cross-sections indicate the presence of well-defined layers. The contrast between phases A and B stems from the polarized light used for these observations, with the A phases loaded with MMT appearing shinier. Nevertheless, even if the individual layers do not have the expected thickness, one can clearly identify the strong reduction in thickness with the increasing number of layers. 

The pole figures of the five samples containing from 1 to 1025 layers for an equivalent thickness of 100 µm were measured, and the corresponding average intensities were calculated and plotted in Figure 11. In each case, the integral breadth was extracted according to Equation (1) (Table 8).

**Figure 13:**
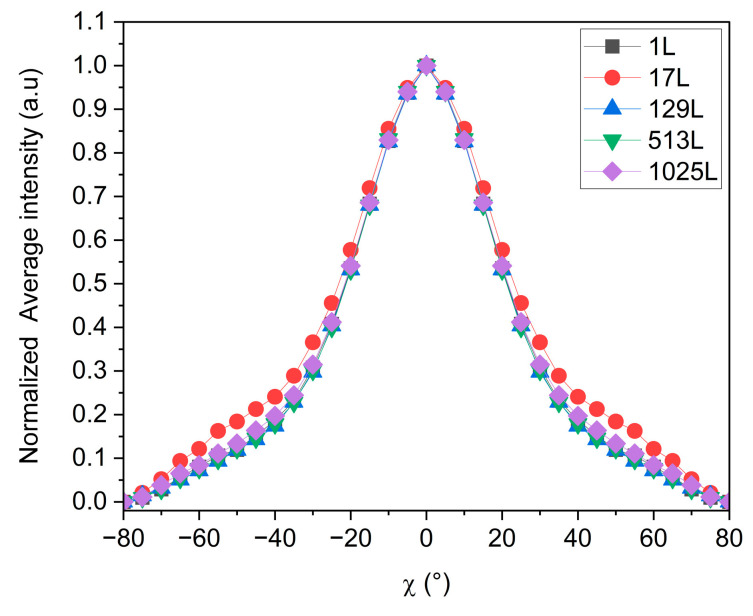
Average intensity over Φ versus χ from 002 MMT pole figures of each sample. Each film has an overall thickness of 100 µm, while the number of layers in the film varies, as indicated in Table 8.

Surprisingly, neither the curves shown on Figure 13 nor the corresponding integral breadths indicate any enhancement of the orientation of the MMT nano-sheets when the thickness of the individual layers was reduced from 100 µm to 98 nm with the increasing the number of layers (Table 8). Indeed, in the TEM images on Figure 7a,b, it can be observed that the montmorillonite nano-sheets embedded in the LLDPE matrix are very flexible, making them potentially more challenging to orient in the plane. This is why, even at a layer thickness of 98 nm, we still cannot achieve controlled orientation.

#### 3.3.2. The Uniaxial Stretching

Another way to reduce the thickness of individual layers significantly consists of applying a uniaxial stretching during film manufacturing. This is achieved by adjusting the take-off speed of the chill roll placed right after the die from which the melted polymer exits in the form of a film. This stretching step results in a reduction in the overall thickness of the film, and therefore in the shrinking of the thickness of the individual layers too [21]. 

**Figure 14:**
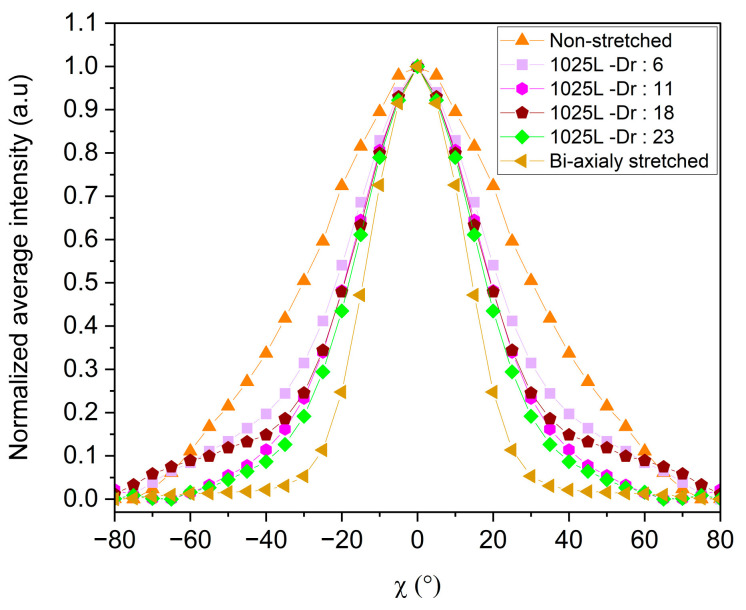
Average intensity over Φ versus χ from 002 MMT pole figures of each sample. Each film has 1025 layers, and the draw ratio varies from 6 to 23.

As mentioned above, previous works have shown that the MNL technology allows us to confine polymer crystal growth and thereby to control the orientation of the grown crystals when the individual layers reach thicknesses of about 30 to 40 nm [53]. In order to verify whether the same effect is observed for MMT embedded in the polymer matrix, we have produced 1025-layer films stretched at various speeds. As indicated in Table 9, this allowed us to reach individual layer thicknesses as thin as 30 nm in the case of the highest stretching speed applied (10 m.mn^−1^). Figure 14 shows the normalized intensity of the XRD signals measured for the 002 Bragg peak of the MMT, in the case of 1025-layer films stretched at various speeds. 

Some improvements in the “in-plane” orientation of the MMT nano-sheets can be noted with the reduction in the thickness of the individual layers. The corresponding integral breadths decrease from 70° with no stretching down to 42° with a draw ratio of 23.

Thus, reducing the layer thickness down to 30 nm led to rather significant improvements in the “in-plane” orientation of the MMT. However, this did not allow us to reach the level of orientation obtained in the bi-axially stretched sample shown in Figure 14. These observations suggest that the LLDPE crystals may play a role in the “in-plane” orientation of MMT nanosheets, as the orientation is largely enhanced when the stretching occurs below the melting temperature of the polymer. 

Indeed, by stretching the molten material, we induced deformation of the sample in a rubbery state, thus promoting elongation and the disentangling of polymer chains to form more oriented structures. By increasing the applied draw ratio on the films, we rapidly solidified these oriented structures, giving them less time to relax; we thus increased the confinement applied to the MMT charges and promoted their orientation [54]. An additional study on the morphology of LLDPE crystals in the samples below shows that increasingly oriented structures are generated with the increase in DR (this aspect will be detailed in another paper). However, these effects on the improvement of MMT orientation are not as significant as in the case of biaxial stretching. Indeed, in the case of biaxial stretching, in addition to being in a rubbery state, crystalline domains are retained and seem to promote the orientation of MMT charges. It can be suggested that these crystalline domains act as nodes, forming a network-like structure between montmorillonite and LLDPE, which appears to facilitate orientation further, as indicated by the work of Ren. et al. [55] 

## 4. Conclusions

In this study, we confirmed that organomodified montmorillonite is more effective than MMT with sodium ion intercalation in reaching a high level of exfoliation in a polyolefin matrix such as LLDPE. We illustrated the importance of combining various characterization methods to achieve an accurate estimate of the achieved level of exfoliation. We have shown that the integral breadth parameter seems suitable for quantifying the orientation of the MMT nano-sheets in the polymer film, and for qualifying the “in-plane” orientation. Our investigations reveal that the combination of the exfoliation level and the MMT concentration does improve the orientation of the MMT in the polymer films. However, to benefit from the advantage of concentration in cast extrusion, it is necessary to find the optimal concentration that maintains good chain mobility and thus promotes better “in-plane” orientation. Surprisingly, we observed that the multi-nano-layer architecture alone does not enhance the “in-plane” orientation of the MMT nano-sheets. However, stretching appeared to be quite an efficient way of aligning the 2D materials parallel to the surface of the film, especially when this operation is carried out below the melting temperature of the polymer matrix. We tentatively attribute this effect to an interaction between the MMT and the LLDPE crystals. All these results pave the way for further investigations of the MMT nano-sheet environment in those samples, and the study of the MMT/polymer morphological interactions. 

## Figures and Tables

**Table 1:** List of the compounds, their composition, and varying processing parameters.

Name	CNa+ Content (wt%)	C20A Content (wt%)	Feed Rate (kg/h)
CNa_20_5	20	_	5
CNa_5_0.5	5	_	0.5
CNa_5_1	5	_	1
CNa_5_3	5	_	3
CNa_5_5	5	_	5
CNa_5_10	5	_	10
C20A_20_5	_	20	5
C20A_5_0.5	_	5	0.5
C20A_5_1	_	5	1
C20A_5_3	_	5	3
C20A_5_5	_	5	5
C20A_5_10	_	5	10
C20A_15_5		15	5
C20A_10_5	_	10	5
LLDPE/LLDPE-g-MA	-	-	5

**Table 2:** List of the films made via thermos-compression.

Name	CNa+ Content (wt%)	C20A Content (wt%)	Feed Rate (kg/h)
CNa_5_5_Thermo Compression	5	_	5
C20A_5_5_ Thermo Compression	_	5	5
C20A_10_5_Thermo Compression	_	10	5
C20A_15_5_Thermo Compression	_	15	5
C20A_20_5_Thermo Compression	_	20	5

**Table 3:** List of the monolayer films made via cast extrusion.

Name	CNa+ Content (wt%)	C20A Content (wt%)	Feed Rate (kg/h)
C20A_5_5_Cast extrusion or 1L	_	5	5
C20A_20_5_ Cast extrusion	_	20	5

**Table 4:** List of the multi-nano-layer films.

Name	Number of LME	Number of Layers	Draw Ratio	Overall Film Thickness
17L	3	17	6	100 µm
129L	6	129	6	100 µm
513L	8	513	6	100 µm
1025L or 1025L–Dr:6	9	1025	6	100 µm
1025L Non-stretched	9	1025	/	800 µm
1025L–Dr:11	9	1025	11	60 µm
1025L–Dr:18	9	1025	18	40 µm
1025L–Dr:23	9	1025	23	30 µm

**Table 5:** Melt yield stress (σ_0_) extracted from the Carreau–Yasuda model (Equation (1)) applied to the curves displayed in Figure 5a on compounds with Na+ montmorillonite.

Sample	σ_0_ (Pa)	σ_0_ Compound/σ_0_ Matrix
CNa^+^_20_5	1659	6.57
CNa^+^_5_0.5	899	3.56
CNa^+^_5_1	633	2.51
CNa^+^_5_3	725	2.87
CNa^+^_5_5	281	1.11
CNa^+^_5_10	231	0.92
LLDPE_LLDPE-g-MA	253	/

**Table 6:** Melt yield stress (σ_0_) extracted from the Carreau–Yasuda model (Equation (1)) applied to the curves displayed in Figure 5b on compounds with C20A montmorillonite.

Sample	σ_0_ (Pa)	σ_0_ Compound/σ_0_ Matrix
C20A_20_5	107,980	427.63
C20A_5_0.5	1530	6.06
C20A_5_1	1890	7.49
C20A_5_3	1741	6.89
C20A_5_5	1823	7.22
C20A_5_10	1612	6.39
LLDPE_LLDPE-g-MA	253	/

**Table 7:** Calculated integral breadth of the peaks in Figure 9a,b.

MMT Content (wt%)	Process	Integral Breadth (°)
5	Thermo–compression	64
	Cast extrusion	52
20	Thermo–compression	48
	Cast extrusion	45

**Table 8:** Individual layer thicknesses calculated based on the number of layers in a 100 µm thick film, and calculated integral breadth of peaks in Figure 13.

Number of Layers	Calculated Individual Layer Thickness	Integral Breadth (°)
1	100 µm	52
17	6 µm	58
129	775 nm	51
513	195 nm	52
1025	98 nm	53

**Table 9:** Individual layer thicknesses calculated based on the overall thickness of the film in a 1025-layer film, and calculated integral breadth of peaks in Figure 14.

Draw Ratio	Calculated Individual Layer Thickness	Integral Breadth (°)
Non-stretched	780 nm	70
6	98 nm	53
11	58 nm	46
18	39 nm	50
23	29 nm	42
Bi-axially stretched	/	30

## Data Availability

Data are contained within the article.
